# Sustainable development and public health: rating European countries

**DOI:** 10.1186/1471-2458-13-77

**Published:** 2013-01-28

**Authors:** Kristina Seke, Natasa Petrovic, Veljko Jeremic, Jovanka Vukmirovic, Biljana Kilibarda, Milan Martic

**Affiliations:** 1 Communication Centre, Institute of Public Health of Serbia “Dr Milan Jovanovic Batut”, dr Subotica, 5, Belgrade, Serbia; 2 Department for Ecology, Faculty of Organizational Sciences, University of Belgrade, Jove Ilica, 154, Belgrade, Serbia; 3 Department for Operations Research and Statistics, Faculty of Organizational Sciences, University of Belgrade, Jove Ilica, 154, Belgrade, Serbia; 4 Department for Marketing Research, Belgrade Business School, Kraljice Marije, 73, Belgrade, Serbia; 5 Centre for Health Promotion, Institute of Public Health of Serbia “Dr Milan Jovanovic Batut”, dr Subotica, 5, Belgrade, Serbia

**Keywords:** Public health, Sustainable development, I-distance method, Rating countries

## Abstract

**Background:**

Sustainable development and public health quite strongly correlate, being connected and conditioned by one another. This paper therein attempts to offer a representation of Europe’s current situation of sustainable development in the area of public health.

**Methods:**

A dataset on sustainable development in the area of public health consisting of 31 European countries (formally proposed by the European Union Commission and EUROSTAT) has been used in this paper in order to evaluate said issue for the countries listed thereof. A statistical method which synthesizes several indicators into one quantitative indicator has also been utilized. Furthermore, the applied method offers the possibility to obtain an optimal set of variables for future studies of the problem, as well as for the possible development of indicators.

**Results:**

According to the results obtained, Norway and Iceland are the two foremost European countries regarding sustainable development in the area of public health, whereas Romania, Lithuania, and Latvia, some of the European Union’s newest Member States, rank lowest. The results also demonstrate that the most significant variables (more than 80%) in rating countries are found to be “healthy life years at birth, females” (r^2^ = 0.880), “healthy life years at birth, males” (r^2^ = 0.864), “death rate due to chronic diseases, males” (r^2^ = 0.850), and “healthy life years, 65, females” (r^2^ = 0.844).

**Conclusions:**

Based on the results of this paper, public health represents a precondition for sustainable development, which should be continuously invested in and improved.

After the assessment of the dataset, proposed by EUROSTAT in order to evaluate progress towards the agreed goals of the EU Sustainable Development Strategy (SDS), this paper offers an improved set of variables, which it is hoped, may initiate further studies concerning this problem.

## Background

It has been widely recognized that sustainable development and public health are intricately connected [1,2]. Achieving sustainable development largely depends on a healthy populace. Consequently, public health represents not only a significant outcome, but a precondition of sustainable development as well. Furthermore, public health and sustainable development are closely interrelated; both emphasize the need to think about the long term, to work in concert with others, and to integrate environmental, social, and economic factors into decision making [3].

Sustainable development is deals with improving the physical, social, and personal quality of individual lives in ways that do not hinder future generations [4,5]. Environments dominated by air pollution or exposure to toxic chemicals, or mitigated by a number of other poor quality of life standards, naturally result in ill effects to one’s health. Sustainable development therefore cannot occur in societies marked by persistent socio-economic inequalities, large scale environmental degradation, or widespread disease [3].

At the global level, the 1992 Rio Earth Summit has been one of the most significant points in establishing an international policy framework for sustainable development [1,6]. One of its main achievements, Agenda 21, includes a chapter dedicated to health, arguing that [7]:

“Health and development are intimately interconnected. Both insufficient development leading to poverty and inappropriate development resulting in overconsumption, coupled with an expanding world population, can result in severe environmental health problems in both developing and developed nations. Action items under Agenda 21 must address the primary health needs of the world’s population, since they are integral to the achievement of the goals of sustainable development and primary environmental care.” (para 6.1)

In this regard, the importance of investing in improvements to health and the environment as a prerequisite for sustainable development has been recognized at the highest decision making level [8].

This paper provides a representation of the situation of European sustainable development in the area of public health. In order to assess this issue in accordance with individual countries, the statistical data was used which is based on a set of sustainable development-public health indicators. In addition, as to evaluate European sustainable development in the area of public health, EUROSTAT’s list of sustainable development-public health indicators was utilized.

Since improvements in the way such indicators are constructed and used are very important research issues [9], sustainable development is evaluated in this paper by implementing a series of public health variables [10] to create a solitary synthesized indicator. As far as can be found, this is the first attempt in the field of sustainable development and public health to quantitatively integrate a greater number of different variables into one value which will represent a subsequent rank.

The methodology used to do so is very similar to propositions made by Ivanovic in his work [11], where he noted that the list of development indicators used in various national or international research institutions are not always identical, and that there is constant controversy concerning the value and importance of one or another of these well-established indicators [12].

## Methods

The overall aim of the Sustainable Development Strategy (SDS) of the European Union is to identify and develop actions to enable it to attain continuous long-term improvement in life quality through the creation of sustainable communities that are able to manage and utilize resources efficiently, to tap the ecological and social innovation potential of the economy, as well as ultimately being able to ensure prosperity, environmental protection, and social cohesion. “Public Health” is one of the seven key challenges identified in the EU’s SDS [13].

According to the EU’s SDS, the overall objective of public health is to promote public health under equal conditions and improve protection against threats to health.

Evaluating progress towards these and other established goals is an integral part of the SDS. Stemming from the fact that the EU Commission constantly develops and improves Sustainable Development Indicators (SDIs), a set of sustainable development indicators has already been developed in order to monitor the Sustainable Development Strategy in the EU. Therein, for the purposes of this paper, the statistical data on sustainable development in the area of public health used here have been collected from EUROSTAT database (updated July 2011) [10].

As to evaluate sustainable development in the area of public health, a dataset consisting of one headline indicator, of six indicators on health and health inequalities, and of five indicators related to determinants of health as proposed by EU Commission and EUROSTAT (see Table 1) has been analysed.

**Table 1:** Public health sustainable development indicators (EU Commission and EUROSTAT)

**Input variables**	**Indicators**	
Healthy life years at birth, females		
Healthy life years at birth, males		
Life expectancy at birth, females	**Headline**	**indicator**
Life expectancy at birth, males		
Death rate due to chronic diseases, females		
Death rate due to chronic diseases, males		
Healthy life years at age 65, females		
Healthy life years at age 65, males		
Life expectancy at age 65, females		
Life expectancy at age 65, males	**Health and health inequalities**	
Suicide death rate, total by age groups^*^		
Suicide death rate, females by age groups^*^		**Actions / explanatory variables**
Suicide death rate, males by age groups^*^		
Self-reported unmet need for medical examination or treatment, first quintile of equalized income		
Self-reported unmet need for medical examination or treatment, second quintile of equalized income		
Self-reported unmet need for medical examination or treatment, third quintile of equalized income		
Self-reported unmet need for medical examination or treatment, fourth quintile of equalized income		
Self-reported unmet need for medical examination or treatment, fifth quintile of equalized income		
Urban population exposure to air pollution by particulate matter	**Determinants of health**	
Urban population exposure to air pollution by ozone		
Proportion of population living in households considering that they suffer from noise		
Serious accidents at work		

^*^(15–19; 50–54; up to 85).

Quite often, the ranking of specific marks is done in such a manner that the process of taking exams, sport competitions, UN participation, Universities ranking, medicine selection, and many other ranking areas can be seriously affected [14-17].

To assess and rank the set of countries in regard to their public health indicators, the I-distance method, which was initially proposed and defined by B. Ivanovic and which has appeared in various publications since 1963, has been used. I-distance is a metric distance in an n-dimensional space [14], which Ivanovic devised to rank countries according to their level of development based on several indicators [12]. In his work, many socio-economic development indicators are considered, yet all of which utilized, as to calculate a single synthetic indicator that shall thereafter represent the rank [14].

For a selected set of variables *X*^*T*^ *= (X*_*1,*_*X*_*2, …,*_*X*_*k*_*)* chosen to characterize the entities, the I-distance between the two entities *e*_*r*_ *= (x*_*1r,*_*x*_*2r,…,*_*x*_*kr*_*)* and *e*_*s*_ *= (x*_*1s,*_*x*_*2s,…,*_*x*_*ks*_*)* is defined as

$$D\left(r,s\right)=\sum_{i=1}^{k}\frac{\left|d_{i}\left(r,s\right)\right|}{\sigma_{i}}\prod_{j=1}^{i-1}\left(1-r_{\mathrm{ji}.12\ldots j-1}\right)$$

where d_i_(r,s) is the distance between the values of variable X_i_ for e_r_ and e_s_; e.g., the discriminate effect of

$$d_{i}\left(r,s\right)=x_{\mathrm{ir}}-x_{\mathrm{is}},i\in \left\{1,\ldots ,k\right\}$$

σ_i_ the standard deviation of X_i_, and r_ji.12.j-1_ is a partial coefficient of the correlation between X_i_ and X_j_, (j < i), [10,13].

The I-distance method itself is constructed from several iterative phases and is recommended to be calculated through the following four steps:

• Calculate the value of the discriminate effect of the variable X_1_ (the most significant variable which provides the largest amount of information on the phenomena that are ranked; i.e., the variable possessing the greatest correlation coefficient with the I-distance value [18].

• Add the value of the discriminate effect of X_2_ not covered by X_1_[19].

• Add the value of the discriminate effect of X_3_ not covered by X_1_ and X_2_[13].

• Repeat this procedure for all variables [20].

In some instances, it is not possible to achieve the same sign mark for all variables in all sets; subsequently, a negative correlation coefficient and a negative coefficient of partial correlation may occur [21,22], which makes use of the square I-distance even more desirable [14]. The square I-distance is given as:

$$D^{2}\left(r,s\right)=\sum_{i=1}^{k}\frac{d_{i}^{2}\left(r,s\right)}{\sigma_{i}^{2}}\prod_{j=1}^{i-1}\left(1-r_{\mathrm{ji}.12\ldots j-1}^{2}\right)$$

In order to rank the entities (in this case, countries), it is necessary to have one entity fixed as a referent in the observing set using the I-distance methodology. The entity with the minimal value for each indicator, a fictive maximal, or an average value entity can be established as the referent entity. The ranking of entities in the set is therein based on the calculated distance from the referent entity [15].

## Results and discussion

In this paper, 31 countries (EU member states, including member states of the last rounds of accession and acceding/candidate countries) have been analysed. To rank these countries, 28 variables have been used (Table 1), whose results are presented in Table 2. According to the I-distance method, the countries of Norway and Iceland head the list. All first listed countries primarily have a significantly high level for the indicator “healthy life years at birth, males” and a very low level of “urban population exposure to air pollution by particulate matter”. Romania, Lithuania, and Latvia rank lowest, having an inadequate level of “healthy life years at birth” and, conversely to first ranked countries, a higher level of “urban population exposure to air pollution by particulate matter”. These countries have a high level of the explanatory variable a “self-reported unmet need for medical examination or treatment”, which may be an indication that citizens in these countries have certain problems in actualizing their rights in the area of health protection. Moreover, very interesting results can be seen for Greece, which, similarly to the lower ranked countries, has very high levels of “urban population exposure to air pollution by particulate matter”, “self-reported unmet needs for medical examination or treatment”, and a high level of “suicide in those up to 85 years of age”. In opposition, Greece has a very high level (similar to leading countries) of “healthy life years at birth”. This indicator is most responsible for this country’s higher position of sixth place in the ranking table (Table 2), which may be attributable to the typical, classic explanations of the country’s Mediterranean diet [23] and living.

**Table 2:** **The results of the I**^**2**^**distance method: I**^**2**^**distance value and rank**

**Rank**	**Countries**	**I**^**2**^**Distance**
1	Norway	64.85
2	Iceland	64.32
3	United Kingdom	64.11
4	Switzerland	61.56
5	Sweden	60.70
6	Greece	59.32
7	Italy	59.22
8	Spain	58.51
9	Netherlands	57.03
10	Ireland	57.02
11	Cyprus	56.49
12	Denmark	55.90
13	Finland	54.78
14	Malta	54.55
15	Belgium	53.76
16	Luxembourg	52.36
17	Germany	51.16
18	France	49.05
19	Austria	48.92
20	Czech Republic	46.84
21	Portugal	46.62
22	Slovenia	46.41
23	Poland	40.48
24	Croatia	39.04
25	Slovakia	38.57
26	Bulgaria	38.29
27	Estonia	33.04
28	Hungary	32.89
29	Romania	32.65
30	Lithuania	32.50
31	Latvia	30.91

With the goal of total comprehension of these rankings and country positions, the input variables were further tested and a coefficient of determination for each variable with its I^2^ distance values was determined. The results are presented in Table 3, according to which, the following variables have proven most significant (more than 80%) in the rating of these countries: “healthy life years at birth, females” (r2 = 0.880), “healthy life years at birth, males” (r2 = 0.864), “death rate due to chronic diseases, males” (r2 = 0.850), and “healthy life years, 65, females” (r2 = 0.844). From the total number of selected variables, nine of them have proven to not be statistically significant in rank forming (p > 0.05). Twenty one selected variables have been demonstrated as being significant in the ranking and evaluation of countries in their sustainable development in the area of public health. Among these, the most significant variable is “healthy life years”. As it appears, among these 21 variables, “urban population exposure to air pollution by particular matter” is the weakest. “Suicide death rates” in younger age groups, “life expectancy at age 65, males”, “serious accidents at work”, “healthy life years at age 65, males”, “urban population exposure to air pollution by ozone”, and the “proportion of population living in households suffering from noise pollution” do not bear any significant impact in the evaluation of countries in their sustainable development in the area of public health.

**Table 3:** **The correlation between I**^**2**^**distance and input variables**

**Variable rank**	**Variables**	**Determination coefficient r**^**2**^	**Sig. (2-tailed)**
1	Healthy life years at birth, females	0.880	**0.000**
2	Healthy life years at birth, males	0.864	**0.000**
3	Death rate due to chronic diseases, males	0.850	**0.000**
4	Healthy life years at age 65, females	0.844	**0.000**
5	Death rate due to chronic diseases, females	0.690	**0.000**
6	Life expectancy at birth, females	0.657	**0.000**
7	Suicide death rate at age 50 to 54, males	0.497	**0.000**
8	Life expectancy at birth, males	0.459	**0.000**
9	Suicide death rate at age 85, males	0.449	**0.000**
10	Suicide death rate at age 85	0.416	**0.000**
11	Suicide death rate at age 50 to 54	0.284	**0.002**
12	Self-reported unmet need for medical examination or treatment, first quintile of equalized income	0.224	**0.007**
13	Self-reported unmet need for medical examination or treatment, fifth quintile of equalized income	0.221	**0.008**
14	Suicide death rate at age 85, females	0.199	**0.012**
15	Self-reported unmet need for medical examination or treatment, second quintile of equalized income	0.185	**0.016**
16	Self-reported unmet need for medical examination or treatment, third quintile of equalized income	0.161	**0.025**
17	Self-reported unmet need for medical examination or treatment, fourth quintile of equalized income	0.158	**0.027**
18	Life expectancy at age 65, females	0.156	**0.028**
19	Urban population exposure to air pollution by particulate matter	0.146	**0.034**
20	Life expectancy at age 65, males	0.089	0.103
21	Serious accidents at work	0.083	0.116
22	Healthy life years at age 65, males	0.080	0.122
23	Suicide death rate at age 15 to 19, males	0.064	0.170
24	Suicide death rate at age 15 to 19	0.061	0.179
25	Suicide death rate at age 50 to 54, females	0.027	0.374
26	Proportion of population living in households considering that they suffer from noise	0.025	0.400
27	Urban population exposure to air pollution by ozone	0.015	0.510
28	Suicide death rate, 15 to 19 - Females	0.004	0.750

Based on the obtained results, one could conclude that the largest influence on the situation of public health for European countries (as one of the key objectives of Sustainable Development Strategy) is to have a country’s lifestyle and living standards, reflected through “healthy life years and life expectancy at birth” variables defined by EUROSTAT’s headline indicator. In addition, variables significant as a reflection of lifestyle and living standards are “death rate due to chronic diseases”, “suicide rate”, and “self-reported unmet need for medical examination or treatment by quintile of equalized income”, all of which are included in EUROSTAT’s health and health inequality indicators. Concerning environmental parameters, according to the results of the I-distance methodology, the only variable influential on the sustainable development of European public health is “urban population exposure to air pollution by particulate matter”.

Established on the abovementioned analyses, the most appropriate set of input variables for the evaluation of sustainable development in the area of public health includes the first thirteen variables (see Table 3) as these variables have the highest impact on the assessment of sustainable public health. The majority of these input variables are included in the headline indicators and health and health inequalities proposed by EUROSTAT. As it can be readily seen in Table 3, the input variables 14–19, have only a minor impact on the assessment of sustainable public health. Furthermore, the input variables 20–28 (see Table 3) make no significant impact on such assessment. Finally, the most noteworthy observation could be that all input variables due to suicide rate in a population younger than 15 years (23, 24, 25, 28 in Table 3) and all other input variables from the group Determinants of health (21, 26, 27 in Table 3), according to applied I distance method, have no impact on sustainable development in the area of public health.

It is sincerely hoped that these inferences will give impulse to more future studies concerning this area.

## Conclusions

Sustainable development is a fundamental goal of the EU, which has been repeatedly enshrined in its treaties since 1997. The EU’s sustainable development strategy brings together many strands of economic, social, and environmental policy under one overarching objective: to continually improve the quality of life and well-being on Earth for present and future generations. At this point in time, when the globe does not yet show clear signs of recovery from the economic and financial crisis, and is facing looming food and energy crises, as well as climate change and threats to social cohesion and security, it is more important than ever to have a coherent and long-term vision for future development [2,24,25].

Public health focuses on the well-being of an entire population rather than on that of the individual [3]. As “traditional” and “modern” hazards are numerous [8], this field must include an array of efforts and labours to maintain the health of all. Furthermore, both sustainable development and public health emphasize long term strategies and objectives, recognizing the need for the integration of environmental, social, and economic factors. Investing in human health is a powerful means to encourage economic growth, protect the environment, and reduce poverty [25]. Most public health investment - for instance, immunization or safe water – bring with them benefits larger than their costs. Moreover, unsustainable production, consumption, and environmental contamination affect public health over the long term. From this point of view, sustainable development should be considered to be a powerful method in improving public health. In the same instance, public health must be observed as a sustainable development precondition, much as Becker had called attention to many years ago in his theory as one component of the stock of human capital [26].

While indicator based assessments may include text, maps, graphics and tables, they are organized around indicators, which – both qualitative and quantitative – enable assessments to be comprehensive while still being selective, covering a wide range of issues to capture human and environmental conditions [27]. In this regard, the I-distance method emphasizes indicators’ quality over their quantity [28].

The possible limitation of the proposed framework could be the selection of indicators. The EU’s official EUROSTAT indicators have been chosen for this analysis precisely for the reason that every ranking statistical method and choice of variables is an essential issue.

The aim of this paper has been to assess the sustainable public health of European countries by applying an I-distance method analysis to them. The results clearly demonstrate that Scandinavian and certain Western European countries lead, due to their high level of living standards. These countries are closely followed by a number of Mediterranean countries, usually typified by their “relaxed” way of life and healthy diet. These results lend credence to the idea that investment in human health, especially in health services, in health promotion, and healthy life styles are a factual and recognizable requisite for sustainable public health.

Furthermore, by the relying on the I-distance method, an optimal set of input variables which are most significant for sustainable development in the area of public health assessment has been proposed. This study suggests that the indicators proposed by EUROSTAT [10] for monitoring sustainable development strategy, variables from Headline indicators, and the set of variables from health and health inequalities be included in the optimal set, while others may be excluded, as they have no significant impact on the assessment of sustainable public health.

## Competing interests

The authors herein declare that they have no conflict of interest pertaining to this work or the information presented thereof.

## Authors’ contributions

KS participated in the design of the study and drafted the manuscript. NP participated in the design of the study and assisted in drafting the manuscript. VJ performed the statistical analysis. JV was involved in the data collection and assisted in performing the statistical analysis. BK participated in the analysis and interpretation of the data. MM assisted in the revising and obtaining of final approval. All authors read and approved the final manuscript.

## Authors’ information

KS graduated from the High Medical School of Professional Studies at the University of Belgrade in 1998 and received her M.Sc. in 2008 from the Faculty of Organizational Sciences, University of Belgrade. At present, she is a Ph.D. student at the same institution. She is currently employed at the Institute of Public Health of Serbia. Her major interests are public health, public health management, social marketing, epidemic intelligence, and communications. NP graduated from the Faculty of Organizational Sciences at the University of Belgrade in 1991, obtained her M.Sc. in 1999, and a Ph.D. in 2002 from the same institution. She currently works as an Associate Professor at the Faculty of Organizational Sciences, University of Belgrade. The area of her scientific research includes: environmental management, sustainable development, environmental education, eco-marketing, environmental design, and public participation in environmental protection. VJ is an Assistant Professor at the Faculty of Organizational Sciences, University of Belgrade, where he acquired his M.Sc. and Ph.D. degrees in Computational Statistics. His major interests are applied statistics, computational statistics, business decision making, and simulation methods. JV is an Assistant Professor at the Belgrade Business School, University of Belgrade, where she acquired her Ph.D. in Statistics and Marketing research. Her areas of research include: applied statistics, marketing research, socio-economic development, and statistical methods in social science. BK is a social medicine specialist, employed at the Institute of Public Health of Serbia. She is a public health PhD student at the Medical Faculty, University of Kragujevac. She has thirteen years of working experience in different public health fields. MM is a Full Professor at the Faculty of Organizational Sciences, University of Belgrade, Serbia where he acquired his M.Sc. and Ph.D. in Operational Research. His major interests are operational research, data envelopment analysis, business decision making, and socio-economic development.

## Pre-publication history

The pre-publication history for this paper can be accessed here:

http://www.biomedcentral.com/1471-2458/13/77/prepub

## References

[B1] Dooris M Baybutt M Connecting Sustainable Development and Public Health: A Critical Exploration with Reference to NHS and Criminal Justice Settings http://www.uclan.ac.uk/schools/built_natural_environment/research/csd/files/Working_Papers_3_Health_Dooris_Baybutt.pdf

[B2] Adshead F Thorpe A Rutter J Sustainable development and public health: a national perspective J R Inst Public Health 2006 120 1102 1105 10.1016/j.puhe.2006.10.003 17097119

[B3] Public Health Agency of Canada Sustainable Development in Public Health 2006 Ottawa: A long term journey begins

[B4] Porritt J Healthy environment-healthy people: the links between sustainable development and health J R Inst Public Health 2005 119 952 953 10.1016/j.puhe.2005.08.004 16185729

[B5] Pluye P Potvin L Denis JL Making public health programs last: conceptualizing sustainability Eval Program Plann 2004 27 121 133 10.1016/j.evalprogplan.2004.01.001

[B6] United Nations Department of Economic and Social Affairs Report of the United Nations Conference on Environment and Development 1992 Rio de Janeiro

[B7] United Nations Agenda 21 Earth Summit the United Nations Programme of Action from Rio 1992 Rio de Janeiro

[B8] Corvalan CF Kjellström T Smith KR Health, Environment and Sustainable Development. Identifying Links and Indicators to Promote Action Epidemiology 1999 10 656 660 10.1097/00001648-199909000-00036 10468446

[B9] Munda G Nardo M Non compensatory/nonlinear composite indicators for ranking countries: a defensible setting Appl Econ 2009 41 1513 1523 10.1080/00036840601019364

[B10] Eurostat European Commission: sustainable development indicators http://epp.eurostat.ec.europa.eu/portal/page/portal/sdi/indicators

[B11] Ivanovic B A method of establishing a list of development indicators STUDY XXIV 1973 Paris: Unesco

[B12] Ivanovic B Fanchette S Grouping and ranking of 30 countries of Sub-Saharan Africa, Two distance-based methods compared 1973 http://unesdoc.unesco.org/images/0000/000063/006393eb.pdf

[B13] Renewed EU Sustainable development Strategy http://register.consilium.europa.eu/pdf/en/06/st10/st10917.en06.pdf

[B14] Radojicic Z Isljamovic S Petrovic N Jeremic V A novel approach to evaluating sustainable development Problemy Ekorozwoju-Problems of Sustainable Development 2012 7 81 85

[B15] Jeremic V Bulajic M Martic M Marovic A Savic G Jeremic D Radojicic Z An evaluation of European countries’ health systems through distance based analysis Hippokratia 2012 16 170 174 PMC3738421 23935275

[B16] Јоvаnоvic М Јеrеmic V Sаvic G Bulајic М Маrtic М How does the normalization of data affects the ARWU ranking? Scientometrics 2012 93 319 327 10.1007/s11192-012-0674-0

[B17] Rаdојicic Z Јеrеmic V Quantity or quality: what matters more in ranking higher education institutions? Curr Sci 2012 103 158 162

[B18] Jeremic V Markovic A Radojicic Z ICT as crucial component of socio-economic development Management 2011 16 60

[B19] Ivanovic B Classification Theory 1977 Belgrade: Institute for Industrial Economic Belgrade

[B20] Jeremic V Seke K Radojicic Z Jeremic D Markovic A Slovic D Measuring health of countries: a novel approach HealthMED 2011 5 1762 1766

[B21] Mihailovic N Bulajic M Savic G Ranking of banks in Serbia YUJOR 2009 19 323 334 10.2298/YJOR0902323M

[B22] Jeremic V Isljamovic S Petrovic N Radojicic Z Markovic A Bulajic M Human development index and sustainability: what’s the correlation? Metalurgia Int 2011 16 63 67

[B23] Tourlouki E Polychronopoulos E Zeimbekis A Tsakountakis N Bountziouka V Lioliou E The ‘secrets’ of the long livers in Mediterranean islands: the MEDIS study Eur J Public Health 2010 20 659 664 10.1093/eurpub/ckp192 19948776

[B24] Eurostat European Commission Sustainable development in the European Union-2011 monitoring report of the EU sustainable development strategy 2011 Luxembourg

[B25] Macroeconomics and health: Investing in health for economic development http://whqlibdoc.who.int/publications/2001/924154550x.pdf

[B26] Becker GS Human capital: A theoretical and empirical analysis with special reference to education 1964 Chicago and London: The University of Chicago Press

[B27] Dalal-Clayton B Bass S Sustainable Development Strategies: A Resource Book 2002 London: Earthscan Publications Ltd

[B28] Singh S Reputation Race in Higher Education is Getting Bigger, but is Getting Better http://forbesindia.com/blog/technology/reputation-race-in-higher-education-is-getting-bigger-but-is-it-getting-better/

